# [Corrigendum] MCM7 amplification and overexpression promote cell proliferation, colony formation and migration in esophageal squamous cell carcinoma by activating the AKT1/mTOR signaling pathway

**DOI:** 10.3892/or.2025.8898

**Published:** 2025-04-16

**Authors:** Yun-Tan Qiu, Wen-Jun Wang, Bing Zhang, Li-Li Mei, Zhi-Zhou Shi

Oncol Rep 37: 3590–3596, 2017; DOI: 10.3892/or.2017.5614

Following the publication of the above article, the authors drew to the Editor's attention that errors had been made in terms of the compilation and assembly of Fig. 4; specifically, regarding the Transwell assay data shown in Fig. 4E, the ‘KYSE510/NS’ and ‘EC9706/Parental’ data panels were overlapping, such that these data appeared to have originated from the same source where the results from differently performed experiments were intended to have been portrayed. The Editorial Office subsequently performed an independent review of the data in this paper, which also revealed that, for the western blots shown in Fig. 5, the ‘KYSE510/AKT1’ and ‘EC9706/mTOR’ protein bands were strikingly similar.

The authors were able to re-examine their original data, and realized how these errors had occurred. The corrected versions of Figs. 4 and 5, now showing replacement data in each case from one of repeated experiments, are shown on the next page. Note that the errors made in assembling the data in these figures did not greatly affect either the results or the conclusions reported in this paper, and all the authors agree with the publication of this corrigendum. The authors regret that these errors went unnoticed prior to the publication of their article, are grateful to the Editor of *Oncology Reports* for granting them this opportunity to publish a corrigendum, and apologize to the readership for any inconvenience caused.

## Figures and Tables

**Figure 4.**
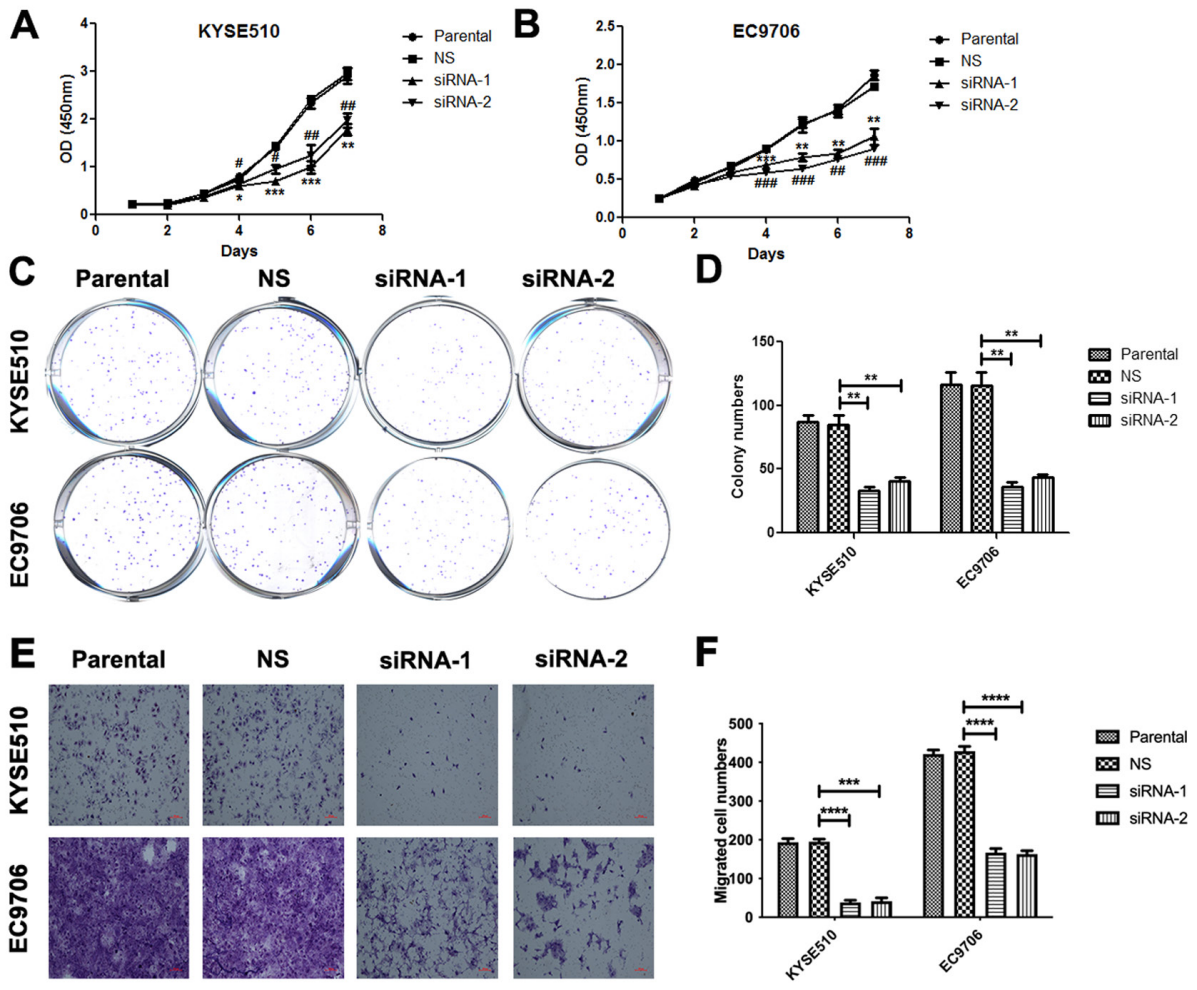
Knockdown of MCM7 suppresses cell proliferation, colony formation and migration of KYSE510 and EC9706 cells. Effects of MCM7 siRNAs on the cell proliferation of KYSE510 (A) and EC9706 (B) cells were detected by Cell Counting Kit-8. (C and D) Effects of MCM7 siRNAs on the colony formation of KYSE510 and EC9706 cells was measured by crystal violet staining. (E and F) Effects of MCM7 siRNAs on the migration of KYSE510 and EC9706 cells were detected by Transwell assay. Representative images are shown. Data are presented as mean ± SEM of n=3 independent experiments. The differences between MCM7 siRNAs group and negative control group were analyzed by using Student's two-tailed t-test. *P<0.05, **P<0.01, ***P<0.001 and ****P<0.0001.

**Figure 5.**
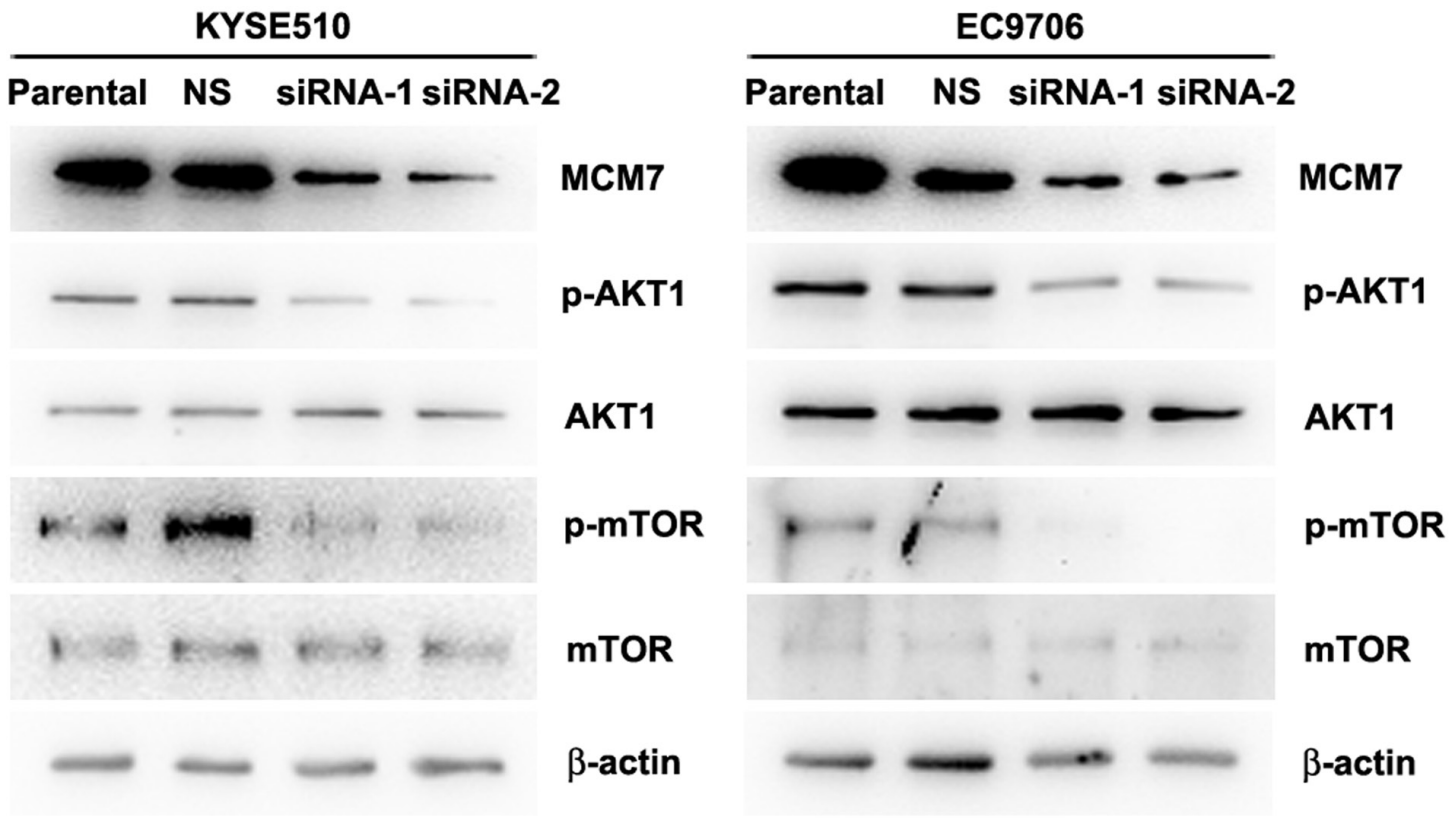
Knockdown of MCM7 inhibits phosphorylation of AKT1 and mTOR in KYSE510 and EC9706 cells. Protein levels of MCM7, p-AKT1, AKT1, p-mTOR, mTOR and β-actin in KYSE510 and EC9706 cells. Cells were transfected with negative control siRNA, MCM7 siRNA-1 and siRNA-2 for 48 h. All the protein levels were determined by western blotting. The experiment was repeated three times.

